# Matricellular Protein CCN5 Gene Transfer Ameliorates Cardiac and Skeletal Dysfunction in *mdx/utrn* (±) Haploinsufficient Mice by Reducing Fibrosis and Upregulating Utrophin Expression

**DOI:** 10.3389/fcvm.2022.763544

**Published:** 2022-04-26

**Authors:** Min Ho Song, Jimeen Yoo, Jae Gyun Oh, Hyun Kook, Woo Jin Park, Dongtak Jeong

**Affiliations:** ^1^College of Life Sciences, Gwangju Institute of Science and Technology, Gwangju, South Korea; ^2^Cardiovascular Research Institute, Icahn School of Medicine at Mount Sinai, New York, NY, United States; ^3^Basic Research Laboratory, Chonnam National University Medical School, Gwangju, South Korea; ^4^Department of Molecular and Life Science, College of Science and Convergence Technology, Hanyang University-ERICA, Ansan, South Korea

**Keywords:** CCN5, DMD, cardiomyopathy, utrophin, AAV9

## Abstract

Duchenne muscular dystrophy (DMD) is a genetic disorder characterized by progressive muscle degeneration due to dystrophin gene mutations. Patients with DMD initially experience muscle weakness in their limbs during adolescence. With age, patients develop fatal respiratory and cardiac dysfunctions. During the later stages of the disease, severe cardiac fibrosis occurs, compromising cardiac function. Previously, our research showed that the matricellular protein CCN5 has antifibrotic properties. Therefore, we hypothesized that CCN5 gene transfer would ameliorate cardiac fibrosis and thus improve cardiac function in DMD-induced cardiomyopathy. We utilized *mdx/utrn* (±) haploinsufficient mice that recapitulated the DMD-disease phenotypes and used an adeno-associated virus serotype-9 viral vector for CCN5 gene transfer. We evaluated the onset of cardiac dysfunction using echocardiography and determined the experimental starting point in 13-month-old mice. Two months after CCN5 gene transfer, cardiac function was significantly enhanced, and cardiac fibrosis was ameliorated. Additionally, running performance was improved in CCN5 gene-transfected mice. Furthermore, *in silico* gene profiling analysis identified utrophin as a novel transcriptional target of CCN5. This was supplemented by a utrophin promoter assay and RNA-seq analysis, which confirmed that CCN5 was directly associated with utrophin expression. Our results showed that CCN5 may be a promising therapeutic molecule for DMD-induced cardiac and skeletal dysfunction.

## Introduction

Duchenne muscular dystrophy (DMD) is an X-linked genetic disorder characterized by progressive muscle weakness. In patients with DMD, a mutation in the dystrophin gene causes non-functional dystrophin proteins. Dystrophin proteins are essential for stabilizing the sarcolemma by connecting cytoskeletal F-actin to the extracellular matrix (1). The pathophysiological consequences of the mutation begin early, with two- to three-year-old patients experiencing a waddling gait and difficulty climbing steps. In their early teenage years, they begin to need wheelchairs and develop respiratory problems (2). Previously, respiratory issues were the most common cause of death in patients with DMD, but improved respiratory support devices have prolonged life expectancy. Currently, cardiac failure is the leading cause of death in patients (1–3). Cardiac fibrosis and LV dilation are observed in approximately 90% of patients over 18 years of age, and progress to severe cardiac dysfunction over time (4, 5).

CCN5, also known as WNT1-inducible signaling pathway protein 2 (WISP-2), is a matricellular protein in the cell communication network (CCN) family. The CCN family is involved in diverse cell behaviors by regulating proliferation, differentiation, and migration (6–9). Our group previously demonstrated that CCN5 prevents cardiac fibrosis (CF) by acting as an antagonist of connective tissue growth factor (CTGF), also known as CCN2, which has been widely studied as a profibrotic molecule (10). We have also shown that CCN5 reverses established cardiac fibrosis by two distinct mechanisms: (1) induction of apoptosis in myofibroblasts (MyoFBs), but not in fibroblasts (FBs) or myocytes, and (2) reverse *trans*-differentiation of MyoFBs to FBs (11).

Adeno-associated viruses (AAVs) are utilized as low-pathogenicity vectors for gene transfer (12). AAV serotypes (AAV.1–9) have different organ specificities; thus, choosing an appropriate vector for high transduction and tissue specificity is necessary. AAV.9 has been shown to be specific to the muscle, heart, liver, lung, and brain (13). Our group previously showed high expression of CCN5 in mouse cardiac tissue when using an AAV.9 vector (11). Other groups have successfully delivered microdystrophin using AAV.9 to the cardiac tissue in *mdx* mice (14).

In this study, we attempted to reduce cardiac fibrosis, which in turn improved cardiac function in a DMD mouse model, through CCN5 gene transfer. Several mouse models have recapitulated the pathophysiology of human DMD (15). Among them, *mdx/utrn* (±) haploinsufficient and *mdx* mouse models are widely used. The *mdx* mouse model has a mild muscular dystrophy phenotype compared to human DMD and a near-normal life span. Conversely, *mdx/utrn* KO mice show extremely early onset of muscle dystrophy and premature death (16). Thus, we chose the *mdx/utrn* (±) mouse model for this study. This model has a total deficiency of the dystrophin gene, but retains half an allele of the autosomal homologous utrophin gene. Thus, it has a relatively normal lifespan, with a relevant degree of muscle dystrophy, fibrosis, and cardiac dysfunction (17–21). Therefore, many groups have used this model and have suggested that it is ideal for testing gene therapies for DMD (17, 19–21). In fact, our characterization of aged *mdx/utrn* (±) mice (>13-month-old) showed a relevant degree of muscular dystrophic phenotype and cardiac dysfunction with severe fibrosis.

In this study, we hypothesized that CCN5 gene transfer may have a therapeutic effect in the context of cardiac function and fibrosis because we previously reported that CCN5 gene therapy reversed pre-established cardiac fibrosis and prevented further deterioration of cardiac function in a TAC-induced heart failure model (11). Moreover, recent *in silico* analysis led us to identify an additional molecular target of CCN5, utrophin. Luciferase assay analysis using the utrophin promoter confirmed that utrophin expression was also significantly restored by CCN5 gene transfer.

In conclusion, we showed in this study that CCN5 gene transfer ameliorates cardiac fibrosis and function not only by the reduction of cardiac fibrosis, but also by the upregulation of utrophin expression. Since utrophin gene transfer has been suggested as an alternative therapy for DMD (22–24), our results provide a new modality to treat severe cardiac dysfunction and fibrosis in patients with DMD.

## Materials and Methods

### Animal Care

All experimental procedures were approved by the Animal Care Committee of the Gwangju Institute of Science and Technology (approval number: GIST-2020-008). C57BL/10ScSn (Dmd*^mdx^*/Utrn*^tm1Jrs^*) mice were purchased from The Jackson Laboratory (Bar Harbor, ME, #016622). We maintained the heterozygous *mdx/utrn* (±) mouse line by mating two male and female heterozygous *mdx/utrn* (±) mice and subsequent genotyping of every generation. To ensure an authentic dystrophic cardiomyopathic model, only aged *mdx/utrn* (±) mice (13–14-month-old; weight, approximately 35 g) were included in our study.

### Genotyping

Genomic DNA was obtained from each mouse tail using the DNeasy Blood and Tissue kit (Qiagen, #69504, MD, United States) following the manufacturer’s protocol. Standard PCR was performed using primers provided by Jackson Lab protocol #27099. Primer 9290: 5′-CTG AGT CAA ACA GCT TGG AAG CCT CC-3′ (*utrn* common reverse); primer 9291: 5′-TTG CAG TGT CTC CCA ATA AGG TAT GAA C-3′ (*utrn* wild type forward); primer 9292: 5′-TGC CAA GTT CTA ATT CCA TCA GAA GCT G-3′ (*utrn* mutant forward). The WT and mut *utrn* PCR product sizes were 646 and 450 bp, respectively. Agarose gel electrophoresis was performed to confirm the individual mouse genotype, and only heterozygote mice, which had two PCR products using the primer sets above, were used in our study.

### AAV.9-CCN5 Production and Injection

Recombinant AAV.9 was produced by the transfection of HEK293T cells. AAV.9 particles suspended in culture media were precipitated with ammonium sulfate and purified by ultracentrifugation on an iodixanol gradient. The particles were concentrated using a Vivaspin centrifugal concentrator (Merck, #Z614645). In this step, iodixanol was diluted using lactated Ringer’s solution *via* multiple washes. The AAV.9 titer was determined by quantitative real-time PCR and SDS-PAGE. AAV.9-Control (Con) and -CCN5 were injected into the tail vein of WT or *mdx/utrn* (±) mice. Mice received 5 × 10^11^ viral genomes and were analyzed 4 weeks later. AAV.9-Con encodes the virus-like particle (VLP) gene, which has no effect on fibrosis or cardiac function. AAV.9-CCN5 encodes the human CCN5.

### Immunohistochemistry

Cardiac tissues from mice were cryopreserved with Tissue-Tek OCT Compound (Sakura, #4583) and sectioned at 6 μm. Tissues were fixed with 4% paraformaldehyde and stained with Masson-Trichrome (Sigma, #HT15-1KT). The fibrotic areas were visualized using a Zeiss Axiophot microscope.

### Fibrotic Area Measurement

Fibrotic tissue was stained blue and non-fibrotic tissue was stained red. The percentage of fibrotic area was calculated as the ratio of the area of fibrosis to the total area of the section using the ImageJ plug-in software (NIH, United States).

### Cardiac Fibroblasts and Myocytes Isolation

Cardiac fibroblasts (FBs) and myocytes were isolated from 13–14-month-old WT or *mdx/utrn* (±) mice. Hearts were immediately resected and retrogradely perfused with a Langendorff system filled with Tyrode’s buffer (137 mM NaCl, 5.4 mM KCl, 1 mM MgCl_2_, 10 mM glucose, 10 mM HEPES, pH 7.4) for 3 min at 37°C. Next, the buffer was switched to an enzyme solution mixture of 250 U/mL type II collagenase (Worthington, # LS004174) and 60 U/mL hyaluronidase (Worthington, # LS002594) and perfused for 20 min. The digested hearts were transferred to a Petri dish with 5% bovine serum albumin containing Tyrode’s buffer. The cardiac tissue was separated and filtered using a 100 μm pore size strainer (Fisher Scientific, # 352098). The filtered fraction was centrifuged at 50 × *g* for 1 min. The supernatant, which contained cardiac FBs, was centrifuged at 250 × *g* for 15 min and resuspended in DMEM (HyClone, # C838R16) containing 10% fetal bovine serum and 1% Penicillin-Streptomycin antibiotics (Thermo Fisher, United States). The cells were seeded in a culture dish; after 4 h, the culture medium was replaced with fresh medium. The precipitated cardiomyocyte fraction was resuspended in phosphate buffered saline and seeded in a 5 μg/mL laminin-coated culture dish. PBS was replaced with M199 medium containing 0.1% bovine serum albumin, 1 × insulin-transferrin-selenium (ITS), 10 mM BDM, 1 × CD lipid (chemically defined lipid concentrate), and 1 × penicillin/streptomycin after 1 h. Cells were incubated at 37°C in a 5% CO_2_ incubator.

### Cardiomyocyte Contractility Analysis and Intracellular Ca^2+^ Transient Measurement

The isolated cardiomyocytes were plated on laminin-coated coverslips. The culture medium was changed to fresh medium after 1 h and incubated at 37°C in a 5% CO_2_ incubator. The next day, cardiomyocytes were treated with 1 μM Fura-2 AM for 10 min before mounting. The cover slip mounted with cardiomyocytes attached was mounted on the stage of an inverted microscope (Nikon Eclipse TE-100F) and perfused (approximately 1 mL/min at 25°C) with Tyrode’s buffer [137 mM NaCl, 5.4 mM KCl, 1 mM CaCl2, 1 mM MgCl2, 10 mM glucose, 0.5 mM Taurin, and 10 mM HEPES (pH 7.4)]. Cardiomyocytes were field-stimulated at a frequency of 1 Hz and 40 V using a stimulator (Grass Technologies, United States). The cardiomyocytes were displayed on a monitor screen using an IonOptix MyoCam camera, and changes in cell length during shortening and lengthening were analyzed using IonWizard software.

A dual-excitation spectrofluorometer (IonOptix Inc., United States) was used to record the fluorescence measurements. The cardiomyocytes, mounted on the stage of an inverted microscope, were exposed to light from a 360 or 380 nm wavelength-filtered 75 W halogen lamp. Fluorescence emissions were detected after initial excitation at 360 nm for 0.5 s and then at 380 nm for the duration of the recording protocol. The 340/380 ratio of the fluorescence intensity (FFI) of the Fura indicated the intracellular Ca^2+^ concentration.

### Echocardiography

Ketamine (100 μg/g) was injected intraperitoneally to anesthetize mice. Short-axis 2D images and M-mode tracings were recorded at the papillary muscle level to measure fractional shortening (FS), left ventricular internal dimension in diastole (LVIDd), and left ventricular internal dimension in systole (LVIDs) (GE Vivid 7 Vision, United States).

### Treadmill and Grip Strength Test

A treadmill test was performed as previously described, with some modifications (25). Prior to beginning measurements, WT and *mdx/utrn* (±) mice were placed on an unmoving treadmill for 2 min for adjustment and then run at 4 m/min for 2 min and 8 m/min for 2 min to warm up. The main exercise was performed at a speed of 12 m/min, until the mice were exhausted. Exhaustion was determined when the mice stopped exercising and did not respond to electrical stimuli for 3–5 s. The aversive electric stimuli were set at 3 Hz, 1.22 mA to maintain the mice running. There was no inclination during this test. The total exercise distance was calculated by multiplying the exercise time and velocity. At the end of the test, mice were allowed to rest for 24 h and the harvested for molecular analysis.

The forearm grip strength of the WT and *mdx/utrn* (±) mice was assessed using an automated grip strength meter (Bioseb, BIO-GS3). The mice grasp a grid with their forepaws instinctively and try to hold while being pulled backward by the tail, releasing when they are unable to maintain grip. Five measurements within 2 min were taken from each animal at 1 week interval after the AAV.9 virus injection.

### Western Blotting

Cardiac tissues, thigh skeletal muscle, and cell lysates were solubilized in RIPA buffer (50 mM Tris, 150 mM NaCl, 0.1% SDS, 1% Triton X-100, pH 8.0) mixed with protease inhibitor cocktail set III (Merck Millipore, #535140). Lysate concentrations were quantified using a Pierce BCA Protein Assay Kit (Thermo Scientific, #23227). Lysates were separated by sodium dodecyl sulfate-polyacrylamide gel electrophoresis (SDS-PAGE) and transferred to polyvinylidene difluoride (PVDF) membranes (Merck Millipore, #IPVH00010). Transferred blots were blocked with 5% non-fat skim milk and incubated with antibodies against mouse monoclonal CCN5 (1:1,000, Sigma, #WH0008839M9), mouse monoclonal α-SMA (1:1,000, Sigma-Aldrich, #A5228), goat polyclonal collagen I (1:1,000, Abcam, ab34710), rabbit polyclonal SERCA2a (1:1,000, a custom antibody from 21st Century Biochemicals), rabbit polyclonal RyR2 (1:1,000, Alomone Lab, ARR-002), rabbit monoclonal NCX1 (1:1,000, Abcam, EPR12739), rabbit polyclonal p-PLN (1:1,000, Badrilla, A010-12AP), mouse monoclonal t-PLN (1:1,000, Badrilla, A010-14), and mouse monoclonal α-tubulin (1:3,000, Santa Cruz, #sc-8035) for 12–16 h at 4°C. After washing the blots with Tris-buffered saline containing 0.1% Tween 20 (TBS-T), they were incubated with secondary antibodies conjugated with horseradish peroxidase (Thermo Fisher Scientific, #31430) and washed again. The band signal was developed using chemiluminescence solution (Millipore, #WBKLS0500). ImageJ plug-in software was used to quantify the western blot results.

### qRT-PCR

The mRNA expression level of utrophin was determined by real-time PCR using the QuantiTect SYBR Green real-time PCR Kit (Qiagen, #204243). Total RNA was isolated from cardiac tissues using TRIzol reagent (Invitrogen, #15596), according to the manufacturer’s instructions. Reverse transcription was performed at 50°C for 20 min, and cDNA was amplified in 20 μL reaction volumes using 10 pmol of primers for 37 cycles: 94°C for 10 s, 57°C for 15 s, and 72°C for 5 s. To calculate the relative abundance of the mRNAs, 18S rRNA was used as an internal control. The primer sequences used in this study were as follows: utrophin, 5′-TCA TGC TAG CCT GGA CCA TTT T-3′ (forward) and 5′-CAC TGA TGG GTG GTT TCC CA-3′ (reverse); 18S rRNA, 5′-TAA CGA ACG AGA CTC TGG CAT-3′ (forward) and 5′-CGG ACA TCT AAG GGC ATC ACA G-3 (reverse).

### Reporter Gene Assay

The utrophin (UTRN) promoter was cloned into a pEZX-PG02.1 vector from a commercial source (GeneCopeia). UTRN promoter DNA (1 μg) and pcDNA3.1-CCN5-myc-his DNA (1 μg) were mixed with 3 μg polyethylenimine (PEI) and incubated for 20 min at 25°C. HEK293T cells were treated with DNA-PEI complexes, and after 4 h the culture medium was replaced with fresh medium. After 72 h, the culture media and lysates were collected and used to detect luciferase activity using a Pierce Gaussia Luciferase assay kit (Thermo Fisher, #16160). The luciferase signal was analyzed using a GloMax microplate reader. *GABP*α DNA was used as a positive control for the UTRN promoter assay.

### Statistical Analysis

Student’s *t*-test and one-way analysis of variance (ANOVA) were used for statistical analyses to determine the significance of the data. Asterisks (*p < 0.05) or double asterisks (^**^p < 0.01) indicate significance. Data in the figures represent the mean ± standard deviation.

## Results

### CCN5 Prevents Cardiac Fibrosis in *mdx/utrn* (±) Mice

**FIGURE 1:**
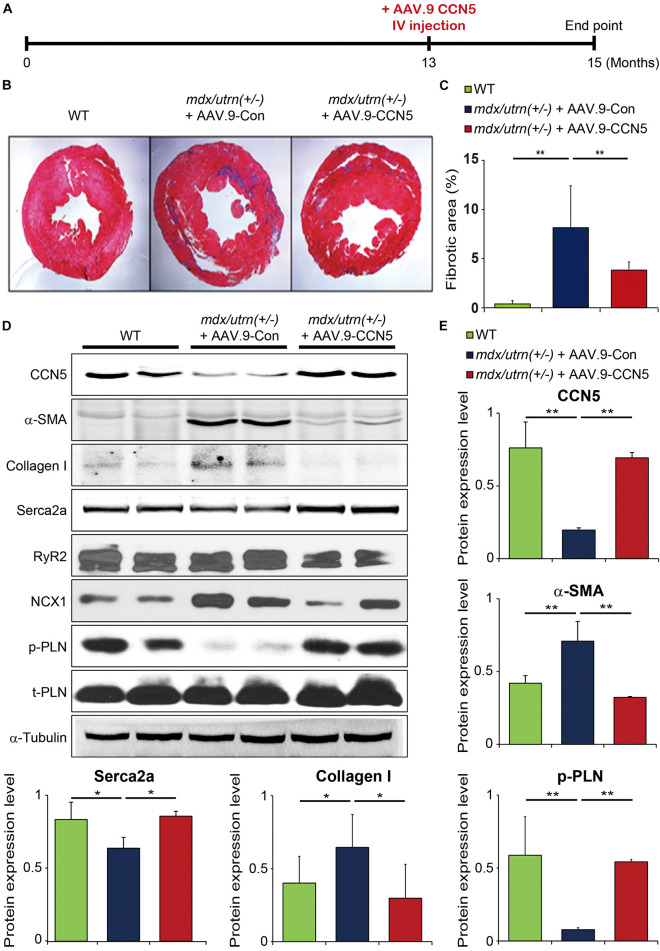
CCN5 prevents cardiac fibrosis in *mdx/utrn* (±) mice. **(A)** Experimental scheme for panels **(B–E)**. *mdx/utrn* (±) mice were injected with AAV.9-Con or CCN5 into the tail vein, and hearts were harvested for experiments 8 weeks later. Age-matched WT mice are shown in comparison. **(B)** Hearts were sectioned and stained with trichrome. Blue areas indicate fibrotic tissue and red areas indicate normal tissue. **(C)** The ratio of fibrotic area over total tissue of the stained hearts was plotted. **(D)** Proteins obtained from cardiac tissue were immunoblotted with antibodies against CCN5, α-SMA, collagen I, SERCA2a, RyR2, NCX1, phosphorylated phospholamban (p-PLN), t-PLN and α-tubulin. **(E)** Protein bands on western blots were scanned and plotted. *n* = 6. *p < 0.05 and **p < 0.01.

Two AAV.9 viral vectors with a CCN5 cassette (AAV.9-CCN5) and control virus (AAV.9-Con) were generated to examine the effects of CCN5 on cardiac fibrosis. We used at least 13-month-old *mdx/utrn* (±) mice to ensure the establishment of cardiac fibrosis and clinical relevance prior to CCN5 gene transfer. Mice received AAV.9-CCN5 (1E11 vg/mouse) through the tail vein and were analyzed 8 weeks later (Figure 1A). First, cardiac tissues were subjected to histological analysis. Heart sections were stained with Masson’s trichrome reagent to analyze CF. Fibrotic areas were significantly developed in the mid- and sub-epicardium within the inferior and lateral walls, in line with the literature (26). AAV.9-Con did not affect fibrotic development (Figures 1B,C). However, a significant decrease in fibrosis within the walls was observed in AAV9-CCN5-administered mice (Figures 1B,C). Molecular signatures of cardiac fibrosis were also evaluated by western blot analysis, which supported this observation. For example, α-SMA, a TGF-β-induced marker of myofibroblast differentiation, was exceptionally inhibited by CCN5 gene transfer (Figures 1D,E). Furthermore, levels of major calcium regulators, such as SERCA2a, RyR2, NCX1, and phospholamban (PLN, phosphor, and total forms) in the heart were measured in the same samples. SERCA2a expression, a hallmark of calcium regulation in the heart, was markedly preserved in CCN5 gene-transfected mice (Figures 1D,E). In addition, phosphorylated PLN levels were significantly restored in the CCN5 gene transfer group. Finally, RyR2 expression was not altered in this model, although NCX1 expression was downregulated in CCN5 injected group. Moreover, the antifibrotic effect of CCN5 was confirmed in isolated cardiac fibroblasts *in vitro* by western blot analysis, and representative profibrotic markers, such as α-SMA, collagen I, and TGF-β, were significantly reduced by CCN5 gene transfer (Supplementary Figures 1A,B). These data prove that CCN5 ameliorates cardiac fibrosis in *mdx/utrn* (±) mice.

### CCN5 Reduces Cardiac Dysfunction in *mdx/utrn* (±) Mice

Restoration of SERCA2a levels results in enhanced calcium handling, which leads to increased cardiomyocyte contractility (27, 28). Therefore, we examined the effect of CCN5 overexpression on cardiomyocyte contractility *in vitro*. We also assessed cardiac function using echocardiography *in vivo*, since cardiac fibrosis negatively affects cardiac function (29–32).

Both cardiac fibroblasts and cardiomyocytes were isolated for further measurements. For this experiment, a modified Langendorff technique was applied to isolate both cell types from the hearts of *mdx/utrn* (±) mice, and Ca^2+^ transients and cardiomyocyte contractility were assessed using the IonOptix system, as described in section “Cardiomyocyte Contractility Analysis and Intracellular Ca^2+^ Transient Measurement.”

**FIGURE 2:**
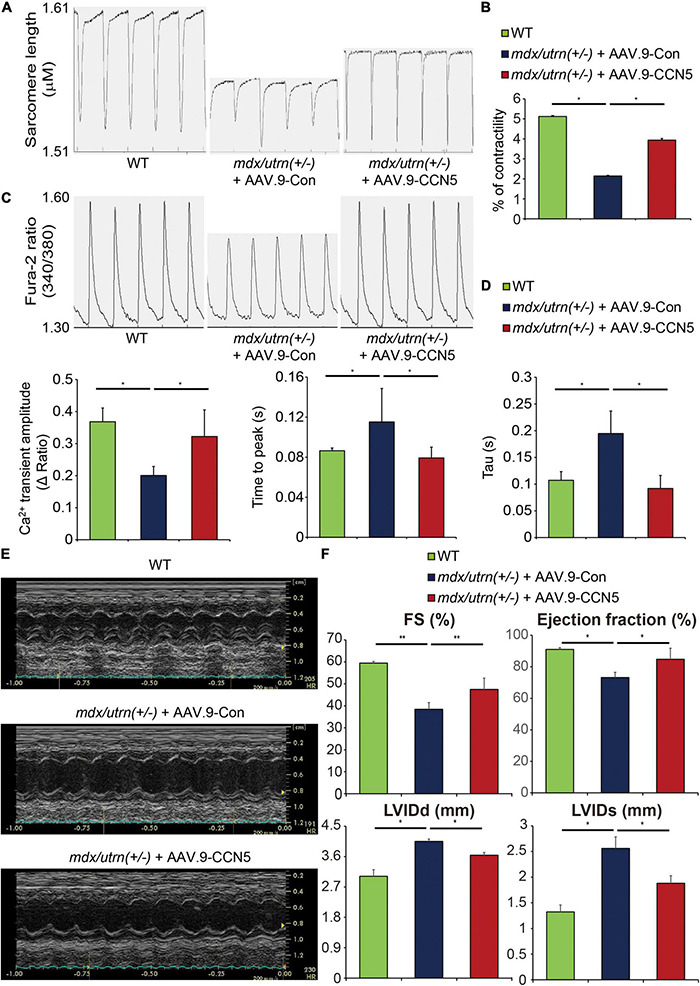
CCN5 reduces cardiac dysfunction in *mdx/utrn* (±) mice. **(A)** The Langendorff system was used to isolate cardiomyocytes. Traces of the sarcomere length were measured in the isolated cardiomyocytes. **(B)** Average contractility was calculated and analyzed. *n* ≥ 40 for analyzing the contractility. **(C)** Traces of the Fura-2 ratio of Ca^2+^ transients were measured in the isolated cardiomyocytes. *n* ≥ 40 for analyzing the Ca^2+^ transient parameters. **(D)** Average Ca^2+^ transient amplitude, time to peak and Tau were calculated and analyzed. **(E)** Representative images of echocardiogram are shown. **(F)** FS, LVIDd, LVIDs and EF were measured to analyze cardiac function by echocardiography. *n* = 6. *p < 0.05 and **p < 0.01.

CCN5 gene transfer resulted in significantly enhanced Ca^2+^ transients and contractility in isolated cardiomyocytes compared to those in control viral vector-delivered mice (Figures 2A–D). Furthermore, the cardiac FB fraction was analyzed by western blotting, and several profibrotic markers were significantly reduced by CCN5 overexpression, as described previously (Supplementary Figure 1).

To investigate *in vivo* cardiac function, echocardiography was performed 8 weeks after gene transfer (Figure 1A). Systolic function indices, including fractional shortening (FS) and ejection fraction (EF), were significantly enhanced, and LVIDd and LVIDs were normalized by CCN5 gene transfer (Figures 2E,F and Supplementary Table 1). These data suggest that CCN5 reduces cardiac dysfunction in *mdx/utrn* (±) mice by restoring calcium regulation and cardiomyocyte contractility both *in vitro* and *in vivo*.

### CCN5 Enhances Exercise Performance of *mdx/utrn* (±) Mice

**FIGURE 3:**
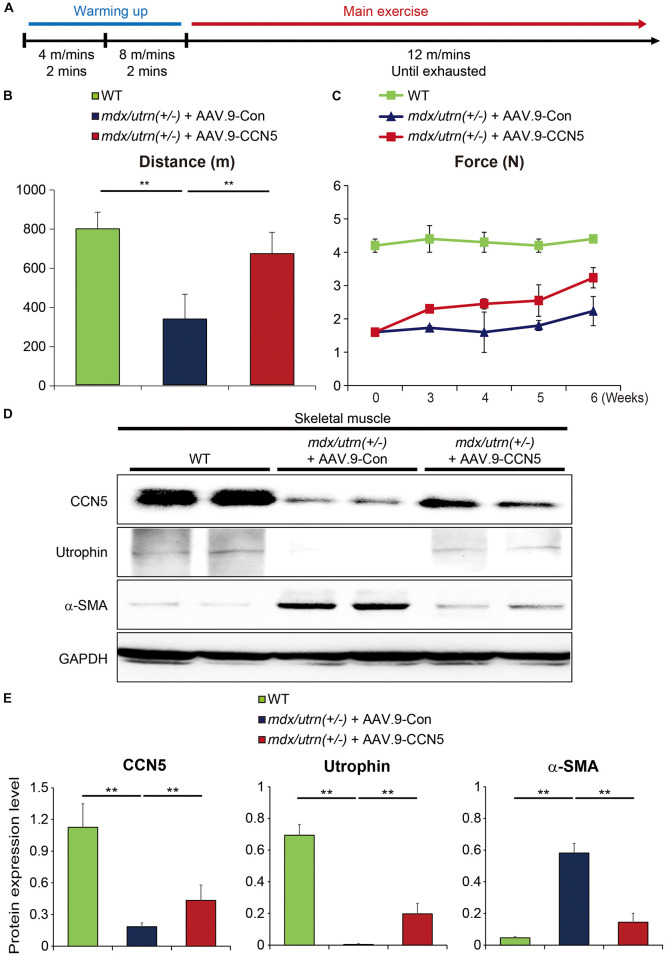
CCN5 enhances exercise performance of *mdx/utrn* (±) mice. **(A)** Experimental scheme for panel **(B)**. Mice were injected with AAV.9-Con or CCN5 into the tail vein, and treadmill tests were performed 8 weeks later. Warming up exercises were serially performed at 4 m/min for 2 min and 8 m/min for 2 min. Main exercise was performed at 12 m/min until the mice were exhausted. **(B)** Total running distance was measured by calculating running time and velocity. **(C)** Comparison of grip strength tests was performed after AAV.9-Con or CCN5 injection. **(D)** Proteins from skeletal muscle tissue were immunoblotted with antibodies against CCN5, utrophin, α-SMA and GAPDH. **(E)** Protein bands on western blots were scanned and plotted. *n* = 6. **p < 0.01.

One of the primary symptoms in patients with DMD is muscle weakness throughout the body, which begins in the lower body around the thighs and pelvis (33–35). In animal models of DMD, muscle weakness has also been observed in multiple reports (36–38). In our aged mouse model, this phenomenon is heightened, and animals are unable to intake food and water properly due to their inability to cling onto the food dispenser. Therefore, food and water were placed at the bottom of the cage to make it easily accessible. However, aged mice with CCN5 gene transfer showed better mobility. Therefore, we performed exercise performance tests using a treadmill and a grip strength instrument for accurate measurements (Figure 3A) (25, 39). *mdx/utrn* (±) mice were exhausted at half the distance compared to the WT control, whereas mice with CCN5 gene transfer exhibited remarkably enhanced running ability (Figure 3B) as well as grip strength (Figure 3C). In addition, molecular signatures analyzed by western blotting and qRT-PCR after CCN5 gene transfer supported these observations. As shown in Figures 3D,E, the transcriptional and translational levels of α-SMA, a myofibroblast marker, were significantly reduced. Surprisingly, utrophin expression was also substantially elevated in skeletal muscle with CCN5 gene transfer, which implies that CCN5 modulates not only tissue fibrosis but also the transcription of utrophin. This result led us to further characterize the relationship between CCN5 and utrophin expression.

### CCN5 Directly Regulates Utrophin Expression

Our results in section “CCN5 Enhances Exercise Performance of *mdx/utrn* (±) Mice” and previous literature imply that CCN5 can act as a transcriptional regulator in the nucleus (Supplementary Figure 3) (40). Our results suggest that CCN5 may modulate gene expression of certain structural molecules in cells. We identified utrophin as a novel target of CCN5 using gene expression profiling (data not shown).

To test this hypothesis, we observed utrophin levels in *mdx/utrn* (±) mice 8 weeks after CCN5 gene transfer. We confirmed *mdx/utrn* (±) genotypes following the Jackson Lab protocol, as described in section “Materials and Methods” (Supplementary Figure 2). We analyzed utrophin expression by western blotting and qRT-PCR.

**FIGURE 4:**
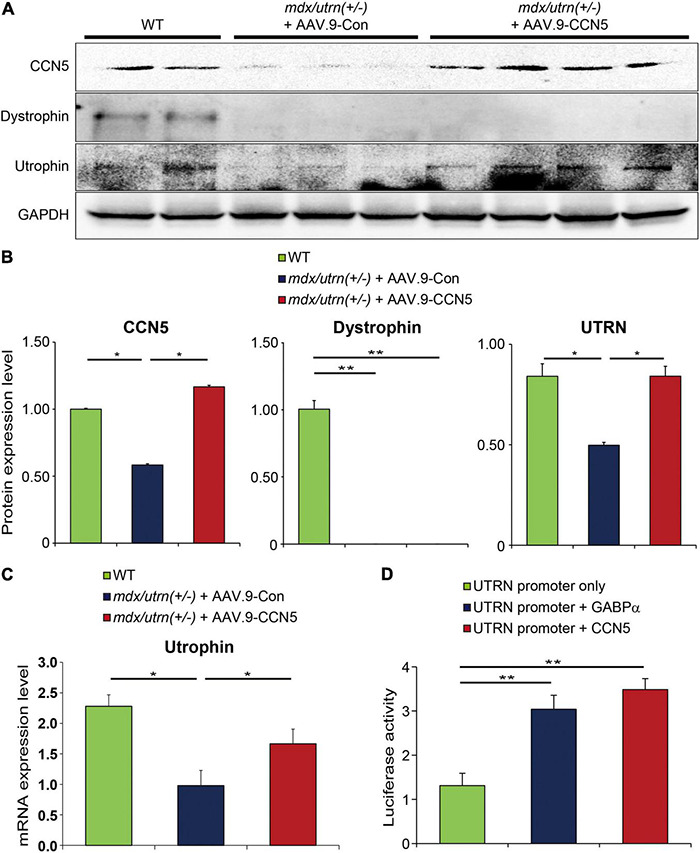
CCN5 directly regulates utrophin expression. **(A)** Proteins obtained from cardiac tissues were immunoblotted with antibodies against CCN5, dystrophin, utrophin and GAPDH. **(B)** Protein bands on western blots were scanned and plotted. **(C)** Relative mRNA expression level of utrophin in hearts. **(D)** Utrophin (UTRN) promoter only, UTRN promoter + GABPα, and UTRN promoter + CCN5 were transfected to 293T cells. Luciferase activity was measured 72 h later. Luciferase activity was analyzed by GloMax Microplate Reader. *n* = 6 for western blots. *n* = 4 for luciferase assay. *p < 0.05 and **p < 0.01.

Utrophin expression was significantly induced at both protein and mRNA levels (Figures 4A–C). In contrast, dystrophin expression was unaltered in all animals (Figures 4A,B). Furthermore, to confirm whether this effect was directly mediated between CCN5 and the target gene promoter, we performed a luciferase assay using the utrophin promoter with the positive control, GABPα, which is a previously reported transcriptional regulator of utrophin (Figure 4D). Our data suggest that CCN5 directly upregulates utrophin expression at the transcriptional level.

## Discussion

Patients with DMD suffer from deficiencies in the functional dystrophin protein, which causes a host of debilitating phenotypes. Dystrophin links actin and glycoproteins in the sarcolemma through the amino acid terminus of each protein. It stabilizes the plasma membrane, which controls the force generated by sarcomere contractions. Deficiency of dystrophin expression causes sarcolemma weakening, functional ischemia, free-radical damage, and cytosolic calcium overload, which promotes muscle damage (1). Therefore, dystrophin is a major target in DMD therapy. In fact, multiple groups have focused on dystrophin as a therapeutic target. Approaches include exon skipping, dystrophin gene transfer using an AAV vector, and Cas9-CRISPR-mediated dystrophin gene editing (41–44). However, the large size of the dystrophin protein, 427 kDa, makes gene therapy difficult because AAV vectors have cargo size limitations.

In addition to dystrophin, utrophin is a potential target for DMD treatment. Utrophin is similar in structure and function to dystrophin, and multiple reports have shown that utrophin compensates for the loss of dystrophin (45–48). In previous studies, utrophin upregulation by small molecules in DMD mouse models successfully ameliorated the symptoms of muscle weakness (49, 50). Since then, many studies have focused on developing novel small molecules that enhance utrophin expression (51). For example, “Ezutromid,” an orally administered small molecule that modulates utrophin production, was clinically tested as a novel DMD treatment despite not meeting the primary endpoint in phase 2 clinical trials (NCT02858362, PhaseOut DMD) (52, 53).

Although DMD patients show excessive fibrosis throughout all muscle fibers in the body, fibrotic pathology has not been the primary focus of treatment. However, significant fibrosis causes stiffness of the muscles and organs, further accelerating muscle deterioration. Cardiac dysfunction is highly correlated with deposition of fibrotic tissues in the hearts of DMD patients (4, 5). Previous studies have suggested that antifibrotic treatments, such as the TGF-β blocker suramin, which prevents muscle fibrosis in *mdx* mice, show promise in preventing DMD-related disease progression (54–56).

Previously, we showed that CCN5 is a promising treatment for cardiac fibrosis in a TAC-induced HF model (11). Therefore, we hypothesized that CCN5 might have a beneficial effect on cardiac fibrosis and function in DMD-associated cardiomyopathy. First, we evaluated whether cardiac fibrosis was reduced by CCN5 gene transfer in *mdx/utrn* (±) mice. As shown by the Masson trichrome tissue staining in Figure 1, cardiac fibrosis was markedly reduced by CCN5 overexpression (Figures 1B,C). The molecular signatures of profibrotic markers and calcium-handling proteins supported our observations (Figures 1D,E). Next, we characterized cardiac function with CCN5 gene transfer *in vitro* and *in vivo* in *mdx/utrn* (±) mice. Cardiomyocyte contractility and calcium transients were measured in cardiomyocytes isolated from the control and CCN5-treated groups. CCN5-treated groups showed significantly enhanced Ca^2+^ transients and contractility compared with the control group. *In vivo* experimental results also showed improved cardiac function, in line with the *in vitro* results.

Our functional data showed that CCN5 improved cardiac contractility and reduced fibrosis. Thus, we attempted to elucidate the mechanism underlying the ionotropic effects of CCN5. We hypothesized that CCN5 downregulated miRNA-25 (miR-25), which then enhanced cardiac contractility. In a previous study, we reported that miR-25 was a major regulator of SERCA2a in a TAC-induced heart failure model (57). Since then, we further characterized the regulatory mechanism of miR-25 biosynthesis and identified the major cell types that produce miR-25. As shown in Supplementary Figure 4, mature miR-25 was dominantly expressed in cardiomyocytes, whereas primary miR-25 was almost exclusively expressed in fibroblasts. This suggests that fibroblasts are the major miR-25 manufacturing cell type. Because CCN5 reverses cardiac fibrosis through the inhibition of transdifferentiation and selectively induces apoptosis in myofibroblasts, miR-25 expression may be downregulated by CCN5 overexpression. In turn, SERCA2a levels were not downregulated by miR-25, thus enhancing contractility and improving calcium handling. Further characterization is necessary to confirm our observations.

In addition, we observed that CCN5 gene transfer enhanced body mobility in *mdx/utrn* (±) mice (Figures 3B,C). We found that utrophin expression was substantially increased in the skeletal muscle of the CCN5-treated group (Figures 3D,E). This result led us to explore the role of CCN5 in the nucleus (Supplementary Figure 3), which has only been hinted at in previous report (40). To characterize the role of CCN5 as a transcriptional regulator, we performed a luciferase assay with a utrophin promoter in HEK-293 cells and compared the results with the *in silico* screening results for potential targets of CCN5 (data not shown). Although any indirect modulation is not completely ruled out, it clearly shows that there is a possible direct interaction between CCN5 and utrophin promoter (Figure 4D). Thus, further experiment such as Chromatin Immunoprecipitation (ChIP) assay will be required to support our observation. Furthermore, cardiac (Figures 4A,B) and skeletal muscle (Figures 3D,E) from CCN5 gene-transferred mice showed significantly enhanced expression of utrophin, strongly supporting our conclusions. Collectively, CCN5 gene transfer not only affects tissue fibrosis and calcium regulation but also modulates utrophin expression at the transcriptional level.

In conclusion, CCN5 is a promising and potent therapeutic molecule for DMD-induced cardiac and skeletal dysfunctions.

## Data Availability Statement

The datasets presented in this study can be found in online repositories. The names of the repository/repositories and accession number(s) can be found in the article/Supplementary Material.

## Ethics Statement

The animal study was reviewed and approved by Institutional Animal Care and Use Committee (IACUC) at the Gwangju Institute of Science and Technology (GIST).

## Author Contributions

WP and DJ conceptualized, designed, and supervised the study. MS and JY performed the experiments and analyzed the data. HK, WP, and DJ analyzed the data. MS, WP, and DJ wrote the manuscript. All authors contributed to the article and approved the submitted version.

## Conflict of Interest

The authors declare that the research was conducted in the absence of any commercial or financial relationships that could be construed as a potential conflict of interest.

## Publisher’s Note

All claims expressed in this article are solely those of the authors and do not necessarily represent those of their affiliated organizations, or those of the publisher, the editors and the reviewers. Any product that may be evaluated in this article, or claim that may be made by its manufacturer, is not guaranteed or endorsed by the publisher.
