# Supplementary material for: Matricellular Protein CCN5 Gene Transfer Ameliorates Cardiac and Skeletal Dysfunction in mdx/utrn (±) Haploinsufficient Mice by Reducing Fibrosis and Upregulating Utrophin Expression
Source: Front Cardiovasc Med. 2022 Apr 26;9:763544. doi: 10.3389/fcvm.2022.763544 (PMC9088811; doi:10.3389/fcvm.2022.763544)
Supplement: Supplementary file 1 [file Data_Sheet_1.PDF]

Supplementary Data

Matricellular protein CCN5 gene transfer ameliorates cardiac and skeletal dysfunction in *mdx/utrn (+/-)* haploinsufficient mice by reducing fibrosis and upregulating Utrophin expression.

Min Ho Song, Jimeen Yoo, Jae Gyun Oh, Hyun Kook, Woo Jin Park, Dongtak Jeong

Supplementary Figure 1. CCN5 gene transfer reduced the expression of pro-fibrotic markers in isolated cardiac fibroblasts.

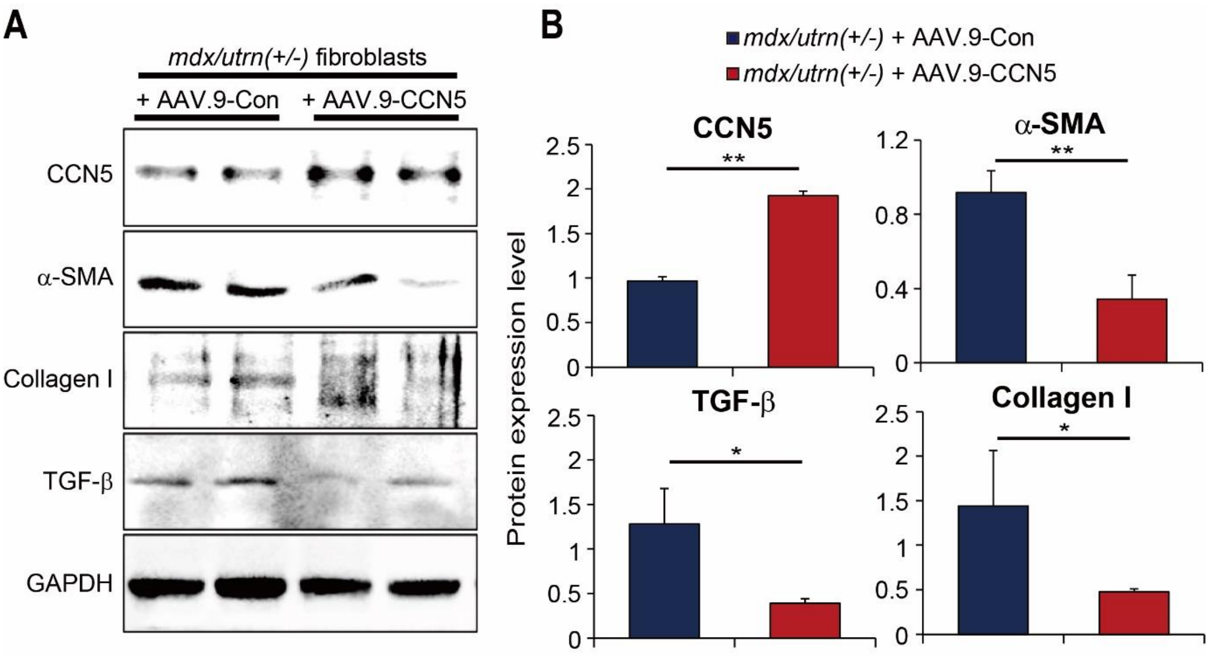

(A) Proteins obtained from cardiac fibroblast of *mdx/utrn (+/-)* mice were immunoblotted with antibodies against CCN5, α-SMA, Collagen I, TGF-β and GAPDH. (B) Protein bands on western blots were scanned and plotted. n=4. *p<0.05, **p<0.01.

Supplementary Figure 2. PCR-based genotype.

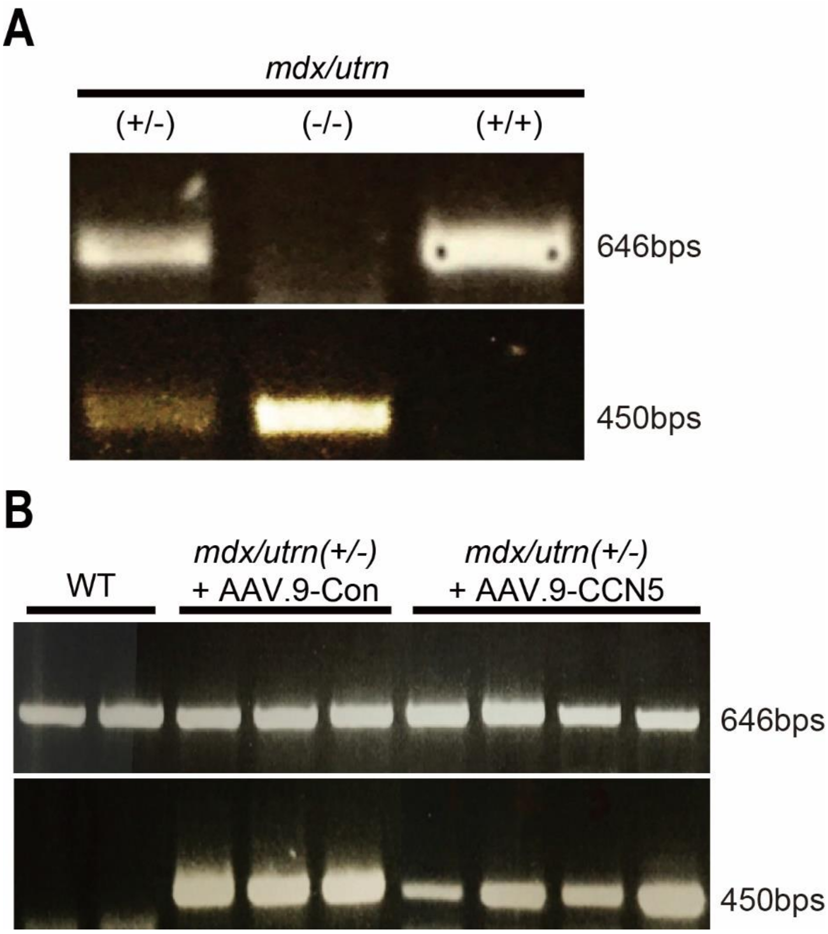

(A) PCR-based genotyping results confirm utrophin alleles of *mdx/utrn (+/-)*, *mdx/utrn (-/-)* and *mdx/utrn (+/+)* mice. (B) PCR-based genotyping results of Figure 4 mice. PCR conditions and the primers used for genotyping were done according to JAX protocol (Jackson Lab, #016622)

Supplementary Figure 3. CCN5 is localized in both cytosol and nucleus.

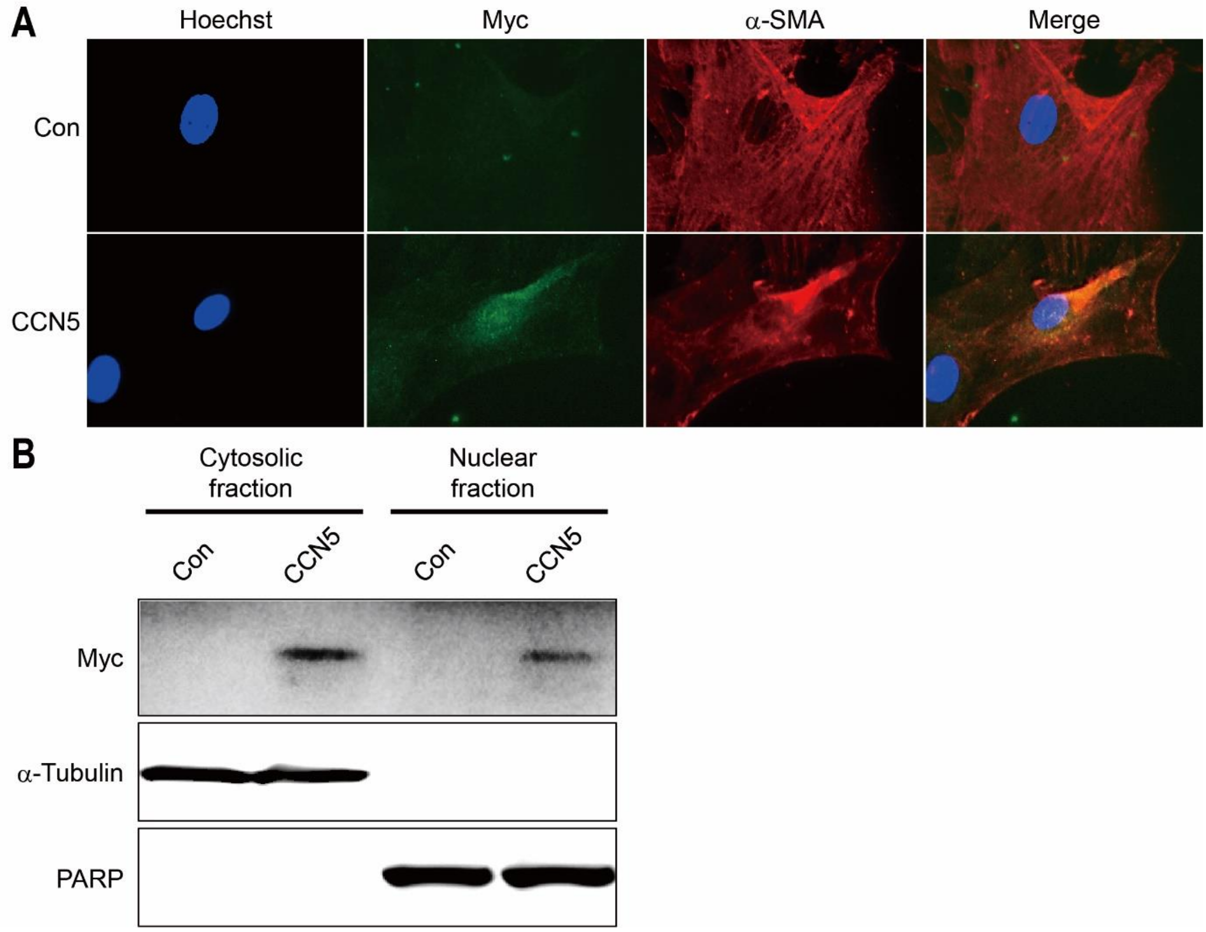

(A) Human cardiac fibroblasts (Lonza, #CC-2904) were treated with TGF-β2 (PeproTech, #100-35B) for 48 hours followed by treatment of 20nM of recombinant human CCN5 protein for 12 hours. Cells were immunostained with anti-Myc (CCN5) and α-SMA antibodies. Nuclei were stained with Hoechst. Anti-rabbit IgG conjugated with Alexa Fluor 488 (Invitrogen, A11008) or anti-mouse IgG conjugated with Alexa Fluor 594 (Invitrogen, A11032) were used as secondary antibodies. (B) Proteins obtained from nuclear fractionation of myofibroblasts were immunoblotted with antibodies against Myc (CCN5), α-Tubulin, and PARP.

Supplementary Figure 4. Cell type-specific expression of primary and mature miR-25 with or without CCN5 gene transfer in the TAC-induced HF mouse model.

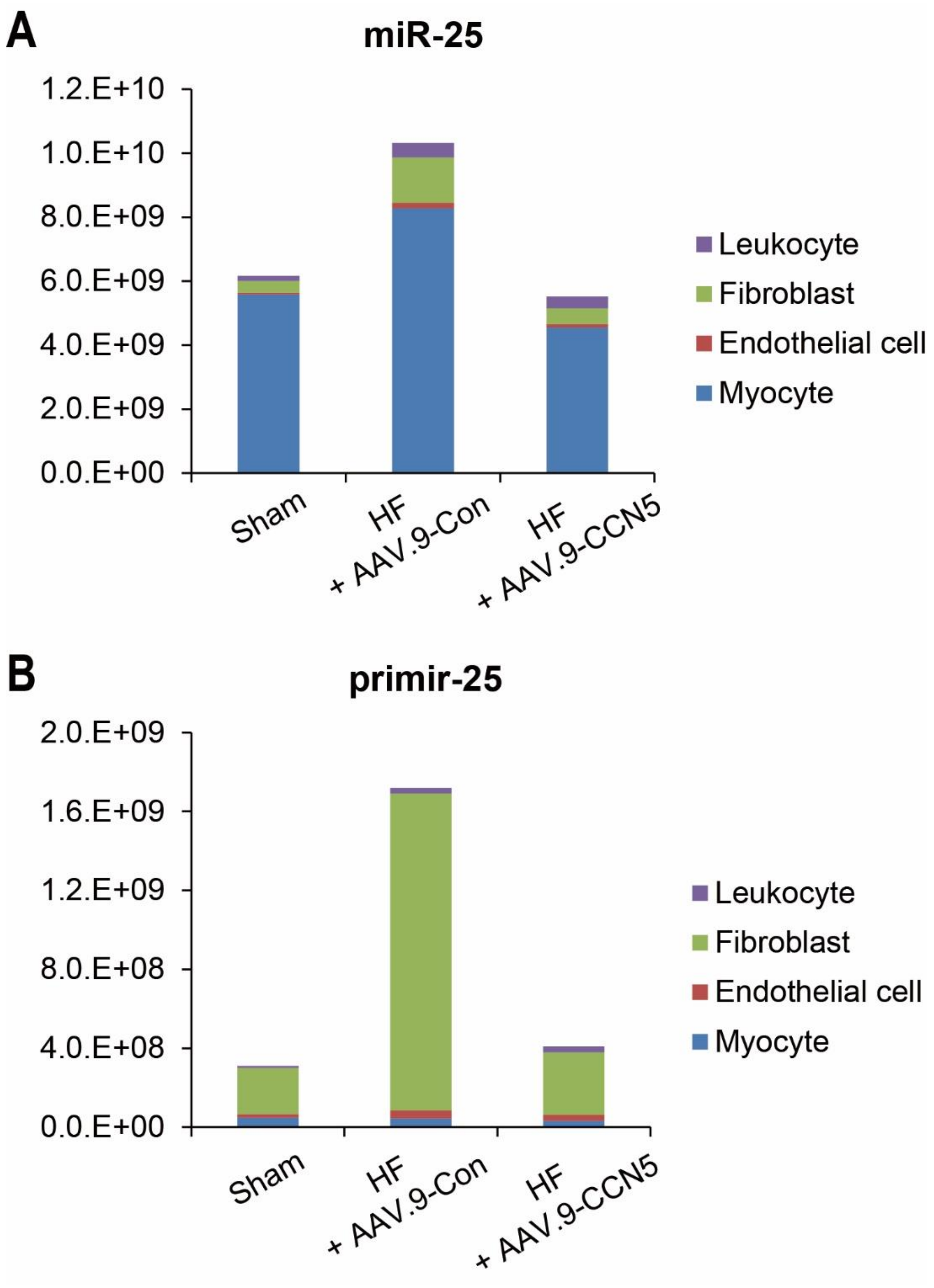

4 different cell types were isolated from the TAC-induced HF mouse model. Endothelial cells were defined by markers $CD31^+$ and $CD45^-$; leukocytes by $CD31^-$ and $CD45^+$; and fibroblasts by $CD31^-$ and $CD45^-$. Non-myocyte fraction from Langendorff-based isolation was stained on ice in PBS containing 6% FCS and 4 mmol/L EDTA using the following antibodies: CD31 (FITC, Bio legend, Cat# 102406, 1:200), CD45 (APC/cy7, BD Biosciences, Cat# 557659, 1:100) or isotype controls IgG2a (BD Biosciences, Cat# 553454). Positive sorting gates for the

CD31 positive or CD45 were set according to unstained controls, isotype controls, and single staining controls. Cell sorting was performed on a BD FACS Aria III (BD Biosciences) with 100 µm nozzle. For qRT-PCR quantification, RNAs were extracted from the sorted cells and applied to be analyzed. (A) The mature miR-25 expression level was analyzed in 4 different cell types isolated from AAV control and CCN5 injected HF mouse model at 3 weeks of post-injection. (B) Using the same mRNA samples, primary miR-25 expression was analyzed in each cell type. n=3. $p<0.05$

Supplementary methods

Real time quantitative PCR for microRNA and gene expression

Total RNA and miRNA-enriched RNA were extracted using the mirVana miRNA isolation kit (Life tech) according to the manufacturer's instructions. cDNAs were synthesized and microRNAs were polyadenylated from total RNA using the qScript (Quanta). qRT-PCR and analysis were performed using an ABI Prism 7500 Real-Time PCR System. The following primers were used for qPCR: miR-25, 5'-CATTGCACTTGTCTCGGTCTGA-3'; pri-mir-25, 5'-CTCACAGGACAGCTGAACACC-3' (Sense), 5'-CCCCCACATCTGCAGTGTTG-3' (Anti-sense).

Fluorescence-activated Cell Sorting (FACS) Analysis.

The non-myocyte fraction from Langendorff-based isolation was stained on ice in PBS containing 6% FCS and 4 mmol/L EDTA using the following antibodies: CD31 (FITC, Bio legend, Cat# 102406, 1:200), CD45 (APC/cy7, BD Biosciences, Cat# 557659, 1:100) or isotype controls IgG2a (BD Biosciences, Cat# 553454). Positive sorting gates for the CD31 positive or CD45 were set according to unstained controls, isotype controls and single staining controls. Cell sorting was performed on a BD FACS Aria III (BD Biosciences) with a 100 µm nozzle. The endothelial cells were defined by the markers $CD31^+$ and $CD45^-$; leukocytes by $CD31^-$ and $CD45^+$; and fibroblasts by $CD31^-$ and $CD45^-$. For fibroblast purity analysis, FACS isolated cells were fixed with 2% PFA, permeabilized and stained in α-SMA (Abcam) or FAP (Abcam) with Vimentin (Abcam). The RNA was extracted from the sorted cells and analyzed.

Table 1. Full parameters for Echocardiography

	WT (n=6)	MDX/UTRN(+/-) Con (n=6)	MDX/UTRN(+/-) AAV.9 CCN5 (n=6)
IVSd (mm)	0.995 ±0.068	0.987 ±0.029	0.940 ±0.005
LVIDd (mm)	3.016 ±0.197	4.048 ±0.073	3.638 ±0.082
LVPWd (mm)	0.961 ±0.085	0.993 ±0.034	0.973 ±0.024
IVSs (mm)	1.717 ±0.096	1.598 ±0.061	1.708 ±0.026
LVIDs (mm)	1.322 ±0.136	2.560 ±0.226	1.880 ±0.349
LVPWs (mm)	1.710 ±0.091	1.598 ±0.061	1.713 ±0.033
EDV(Teich) (mL)	0.072 ±0.013	0.156 ±0.013	0.122 ±0.008
ESV(Teich) (mL)	0.007 ±0.002	0.040 ±0.008	0.019 ±0.010
EF(Teich) (%)	91.014 ±1.127	73.161 ±3.371	84.752 ±7.056
FS (%)	56.251 ±1.858	37.581 ±2.730	48.402 ±8.403
Time (sec)	103.333 ±5.689	102.750 ±4.992	107.500 ±4.007
HR (1/min)	556.219 ±6.949	609.869 ±3.600	567.308 ±8.418
CO(Teich) (L/min)	0.038 ±0.008	0.067 ±0.005	0.058 ±0.001
BW (g)	25.875 ±2.235	32.500 ±1.747	33.150 ±0.354
